# A probability model for predicting *BRCA1* and *BRCA2* mutations in breast and breast-ovarian cancer families

**DOI:** 10.1054/bjoc.2000.1626

**Published:** 2001-03

**Authors:** P Vahteristo, H Eerola, A Tamminen, C Blomqvist, H Nevanlinna

**Affiliations:** Department of Obstetrics and Gynaecology, Helsinki University Central Hospital, PO Box 140 (Haartmaninkatu 2), FIN-00029, Finland; Department of Oncology, Helsinki University Central Hospital, PO Box 180 (Haartmaninkatu 4), FIN-00029, Finland; Department of Oncology, Uppsala University Hospital, Uppsala, S-75185, Sweden

**Keywords:** breast cancer, ovarian cancer, *BRCA1*, *BRCA 2*, mutation, probability model

## Abstract

Germline mutations in *BRCA1* and *BRCA2* genes predispose to hereditary breast and ovarian cancer. Our aim was to find associations between the clinical characteristics and positive mutation status in 148 breast cancer families in order to predict the probability of finding a *BRCA* mutation in a family. Several factors were associated with mutations in univariate analysis, whereas in multivariate analysis (logistic regression with backward selection) only the age of the youngest breast cancer patient and the number of ovarian cancer cases in a family were independent predictors of *BRCA* mutations. A logistic model was devised to estimate the probability for a family of harbouring a mutation in either *BRCA1* or *BRCA2*. Altogether, 63 out of 148 families (43%) and 28 out of 29 (97%) mutation carrier families obtained probabilities over 10%. The mean probability was 55% for mutation-positive families and 11% for mutation-negative families. The models by Couch et al (1997) and Shattuck-Eidens et al (1997) previously designed for *BRCA1* were also tested for their applicability to distinguish carrier families with mutations in either gene. The probability model should be a useful tool in genetic counselling and focusing the mutation analyses, and thus increasing also the cost-effectiveness of the genetic screening. © 2001 Cancer Research Campaign http://www.bjcancer.com


British Journal of Cancer (2001) 84(5), 704–708
© 2001 Cancer Research Campaign
doi: 10.1054/ bjoc.2000.1626, available online at http://www.idealibrary.com on IDEAL

http://www.bjcancer.com

### A probability model for predicting *BRCA1* and *BRCA2* mutations in breast and breast-ovarian cancer families

**P Vahteristo[1], H Eerola[1,2], A Tamminen[1], C Blomqvist[2,3] and H Nevanlinna[1]**

[1]Department of Obstetrics and Gynaecology, PO Box 140 (Haartmaninkatu 2), FIN-00029 Helsinki University Central Hospital, Finland; [2]Department of Oncology, PO Box 180 (Haartmaninkatu 4), FIN-00029 Helsinki University Central Hospital, Finland; [3]Department of Oncology, Uppsala University Hospital, S-75185 Uppsala, Sweden

**Summary** Germline mutations in *BRCA1* and *BRCA2* genes predispose to hereditary breast and ovarian cancer. Our aim was to find associations between the clinical characteristics and positive mutation status in 148 breast cancer families in order to predict the probability of finding a *BRCA* mutation in a family. Several factors were associated with mutations in univariate analysis, whereas in multivariate analysis (logistic regression with backward selection) only the age of the youngest breast cancer patient and the number of ovarian cancer cases in a family were independent predictors of *BRCA* mutations. A logistic model was devised to estimate the probability for a family of harbouring a mutation in either *BRCA1* or *BRCA2*. Altogether, 63 out of 148 families (43%) and 28 out of 29 (97%) mutation carrier families obtained probabilities over 10%. The mean probability was 55% for mutation-positive families and 11% for mutation-negative families. The models by Couch et al (1997) and Shattuck-Eidens et al (1997) previously designed for *BRCA1* were also tested for their applicability to distinguish carrier families with mutations in either gene. The probability model should be a useful tool in genetic counselling and focusing the mutation analyses, and thus increasing also the cost-effectiveness of the genetic screening. © 2001 Cancer Research Campaign http://www.bjcancer.com

**Keywords**: breast cancer; ovarian cancer; *BRCA1*; *BRCA 2*; mutation; probability model

Mutations in the two breast-ovarian cancer susceptibility genes, *BRCA1* and *BRCA2*, account for a varying fraction of breast cancer families in different populations (Szabo and King, 1997). Both *BRCA1* and *BRCA2* mutations are scattered throughout the large coding regions of the genes (Breast Cancer Information Core). In admixed populations, most mutations appear uniquely in single families only, making the mutation screening laborious and expensive. Furthermore, there is also evidence of other predisposing genes (Ford et al, 1998; Kainu et al, 2000). It is, therefore, important to find the clinical risk factors that could best predict the presence of *BRCA1* and *BRCA2* mutations, so that the screening could be directed to potential mutation carrier families.

Several probability models for mutation detection have been developed. These are, however, based only on *BRCA1* (Berry et al, 1997; Couch et al, 1997; Shattuck-Eidens et al, 1997), focus on specific founder mutations in the Ashkenazi population (Foulkers et al, 1999; Hodgson et al, 1999; Hopper and Jenkins, 1999), or require information such as penetrance estimations not available in all populations (Berry et al, 1997; Parmigiani et al, 1998; Chang-Claude et al, 1999).

Here we have developed a model for predicting the presence of a *BRCA1* or *BRCA2* mutation in families with 3 or more relatives affected with breast or ovarian cancer. We also compared this model with those of Shattuck-Eidens et al (1997) and Couch et al (1997) originally designed for *BRCA1* only. Additionally, the frequency of *BRCA1/2* mutations was studied in 295 families with two affected family members to evaluate the feasibility of genetic screening in families with moderate family history.

*Received 26 June 2000*
*Revised 13 November 2000*
*Accepted 14 November 2000*

*Correspondence to:* H Nevanlinna

### PATIENTS AND METHODS

The cohort studied consisted of 148 families with 3 or more 1st or 2nd degree relatives affected with breast or ovarian cancer. The families were identified by patient interviews, and full pedigrees were constructed with the confirmation of all genealogy data through the Finnish population registration as well as diagnostic data through hospital records and/or Finnish Cancer Registry as previously described (Vehmanen et al, 1997a,b; Eerola et al, 2000). Additionally, 295 breast cancer cases with one 1st degree relative affected with breast or ovarian cancer and identified in the patient cohorts described in Eerola et al (2000) were also studied. In the following, these are called small families. The family history of these cases was based on information reported by the index patient. All patients participating in the study signed an informed consent before the blood sample for the genetic analysis was taken. This study has been approved by the Ethical Committees of Departments of Obstetrics and Gynaecology, and Oncology, HUCH, and appropriate permissions were obtained from the Ministry of Social Affairs and Health in Finland.

The mutations identified by a complete mutation analysis of the whole coding sequences and exon/intron boundaries of the genes in 95 of these families have been previously reported (Vehmanen et al, 1997a,b). For 53 other families, all previously reported 18 Finnish *BRCA1* and *BRCA2* mutations (Vehmanen et al, 1997a,b; Huusko et al, 1998; Sarantaus et al, 2000), and one recently discovered new *BRCA1* mutation (3264 delT) were analysed by allele-specific oligonucleotide (ASO) (Friedman et al, 1995) hybridization or restriction fragment length polymorphism (RFLP). The RFLP analyses were designed such that incomplete digestion would lead to a false positive hence minimizing the possibility of a false negative result. Sequences of the PCR primers and ASO probes, as well as the enzymes used for digestions are available upon request. Protein truncation test (PTT) (Hogervorst et al, 1995; Håkansson et al, 1997) of *BRCA1* exon 11 and *BRCA2* exons 10 and 11 was also used to search for new mutations in 36 families with an ovarian cancer case or a breast cancer patient diagnosed below 50 years. All positive mutation detection results were confirmed by direct sequencing using an ABI PRISM 310 Genetic Analyser and Dye Terminator Cycle Sequencing Ready Reaction Kit according to the manufacturer's instructions (PE Applied Biosystems, Foster City, CA, USA).

For 295 small breast cancer families ASO and RFLP analyses were used to screen all known Finnish mutations, and direct sequencing was used to confirm the positive screening results. In previous studies, 11 recurrent founder mutations have been found to account for vast majority (84%) of all Finnish *BRCA1* and *BRCA2* families (Vehmanen et al, 1997a,b; Huusko et al, 1998). Therefore, screening of the known mutations was used to evaluate the feasibility of screening of the *BRCA1* and *BRCA2* genes in these families.

### Statistical analysis

Associations between specific familial characteristics (presented in Table 1) and the presence of a *BRCA1* or *BRCA2* germline mutation were studied by univariate and multivariate analyses. For univariate analysis, Mann-Whitney and Fisher's exact tests (SPSS 8.0 for Windows) were used. Variables that were predictive of a mutation in a univariate analysis were used in a multivariate analysis (stepwise backward logistic regression, 99%), and based on that a logistic probability model for harbouring a deleterious mutation was devised.

The models by Couch et al (1997) and Shattuck-Eidens et al (1997), previously designed for estimating mutation probability in the *BRCA1* gene, were also tested in the 148 families and compared to the model developed here for their applicability to distinguish carrier families with mutations in either gene.

### RESULTS AND DISCUSSION

### Mutations identified

A total of 29 germline mutations was found in 148 families (19.6%), 16 in *BRCA1* (10.8%) and 13 in *BRCA2* (8.8%). In addition to previously known Finnish mutations, two new protein truncating mutations were identified (*BRCA1*, 1806 C $\rightarrow$ T and *BRCA2*, 5797 G $\rightarrow$ T). Both of these mutations were subsequently found also in other study cohorts (Syrjäkoski et al, 2000; Sarantaus L, personal communication) making the total number of recurrent mutations in Finland now 13. Altogether, 24 (86%) of the mutation-positive patients carried one of the recurrent mutations, and 5 patients unique mutations not found in other families so far in Finland.

### Factors associated with positive mutation status

Several factors were associated with the presence of germline *BRCA1* or *BRCA2* mutations in the univariate analysis (Table 1). In the multivariate analysis, only two variables were still significant: the number of ovarian cancer cases in a family ($P < 0.00005$) and the age at diagnosis of the youngest breast cancer patient ($P$ = 0.0007). The presence of breast and ovarian cancer in the same patient was not significant in multivariate analysis, probably because it is closely associated with ovarian cancer cases overall. Bilateral breast cancer, another factor that has been correlated with a positive mutation status by for example Shattuck-Eidens et al (1997) and Ligtenberg et al (1999), was not significant in univariate analysis and, therefore, not included in further analysis.

Families carrying a mutation in either *BRCA1* or *BRCA2* were also analysed separately (data not shown). The results were similar for both genes except for the number of breast cancer patients that was associated with a *BRCA2* mutation status in the univariate analysis. In the multivariate analysis the same variables were significant for both genes and, therefore, one common model could be used for distinguishing all mutation carriers. Early age of breast cancer onset as well as the presence of ovarian cancer in a family are thus highly characteristic for Finnish *BRCA2* families also. It is of interest to note that only one of the *BRCA2* mutations in this study was in the OCCR region where a higher risk of ovarian cancer, relative to breast cancer, has been suggested (Gayther et al, 1997; Ford et al, 1998).

### Probability of identification of a mutation in the family

Based on the results from the multivariate analysis, a probability model for harbouring a deleterious mutation was devised, and can be written in the form of:

**Table 1** Variables tested and the associations found in univariate analysis

| | *BRCA1 / BRCA2* | non-*BRCA1/2* | P value in univariate analysis |
|---|---|---|---|
| Variables concerning the number of breast and ovarian cancer cases | Mean number of cancer cases | | |
| Mean number of breast cancer cases in a family | 3.5 | 3.8 | 0.304 |
| Mean number of ovarian cancer cases in a family | 1.4 | 0.2 | <0.0005 |
| Mean number of bilateral breast cancer cases in a family | 0.5 | 0.3 | 0.292 |
| Variables concerning the age at diagnosis | Age in years | | |
| Age at diagnosis of the index case | 41.3 | 51.4 | <0.0005 |
| Age at diagnosis of the youngest breast cancer patient | 38.5 | 46.0 | <0.0005 |
| Age at diagnosis of the youngest ovarian cancer patient | 52.0 | 59.7 | 0.056 |
| Mean age at diagnosis of the breast cancer cases | 47.6 | 56.4 | <0.0005 |
| Variables concerning the presence of different cancer types | Proportion | | |
| Presence of ovarian cancer in a family | 79% (23/29) | 20% (24/119) | <0.0005 |
| Presence of breast and ovarian cancer in the same individual | 34% (10/29) | 2.5% (3/119) | <0.0005 |
| Presence of bilateral breast cancer in a family | 31% (9/29) | 24% (29/119) | 0.482 |
| Presence of prostate cancer in a family | 24% (7/29) | 15% (18/119) | 0.272 |

$$p = e^L / (1 + e^L)$$

and L can be calculated from the equation $L = 2.87 + (–0.14) \times V_1 + 2.11 \times V_2$ where 2.87 is a constant and –0.14 and 2.11 are the coefficients received from the regression analysis, $V_1$ is the age of the youngest breast cancer patient in a family, and $V_2$ is the number of ovarian cancer cases in a family.

Among the 148 study families, 97% (28/29) of the mutation carrier families obtained a probability greater than an arbitrary cut off value of 10%. The mean probability was 55% for mutation-positive families and 11% for mutation-negative families. Altogether, out of 148 families 63 (43%) obtained probabilities over 10% and among these, 28 (44%) were mutation carrier families. Thus by using this model, mutation screening could be directed to a significantly smaller proportion of families.

Similar results were obtained also with the models of Shattuck-Eidens et al (1997) and Couch et al (1997) originally designed for *BRCA1* (Table 2). Thus these models distinguish also *BRCA2* mutation carrier families very efficiently. The one mutation-positive family missed in all 3 models has 3 affected breast cancer patients all diagnosed at later age. The proportion of mutations found is higher in the model developed in this study since it has been designed particularly for this study cohort, and the determination of sensitivity as well as specificity of this model requires analysis of a separate test population. The model here was also designed to estimate the carrier probability of a family with 3 or more affected cases, and therefore it could not be extrapolated to cases with a less profound family history.

### Mutation frequencies in families with defined family history of cancer

All families classified by the family history of breast and ovarian cancer as well as age of breast cancer onset (below 40 years) are presented in Table 3. By analysing mutation-positive and -negative families, initially chosen by the criterion of at least 3 breast or ovarian cancer patients among 1st or 2nd degree relatives, we noted that mutation carrier families could be identified by a simple criterion of a breast cancer case diagnosed before the age of 40 or an ovarian cancer case in the family. Altogether, 80/148 (54% of all) families fulfilled this criterion, and among these, 28/29 (97%) of the mutations could be found. This simple criterion alone could thus be used as a rough estimation of a high likelihood of carrying a mutation in such families.

No mutations were found in 21 families with 4 or more cases of breast but no ovarian cancer or young breast cancer patient (diagnosis below 40 years). This is in agreement with our results from

**Table 2** Comparison of the different probability models

| | Shattuck-Eidens | Couch | This study |
|---|---|---|---|
| Mutation positive families identified (total) | 27/29 (93%) | 25/29 (86%) | 28/29 (97%) |
| *BRCA1*-positive families identified | 15/16 (94%) | 14/16 (88%) | 16/16 (100%) |
| *BRCA2*-positive families identified | 12/13 (92%) | 10/13 (77%) | 12/13 (92%) |
| Number of families with the probability >10% | 67/148 (45%) | 42/148 (28%) | 63/148 (43%) |
| Mean probability for *BRCA 1/2*-carriers | 53% | 41% | 55% |
| Mean probability for *BRCA 1*-carriers | 50% | 41% | 59% |
| Mean probability for *BRCA 2*-carriers | 55% | 40% | 50% |
| Mean probability for non-*BRCA 1/2*-carriers | 12% | 7% | 11% |

**Table 3** Family history of breast and ovarian cancer of the families studied

| | Total number of families | Number of mutations | | | Mutation % |
|---|---|---|---|---|---|
| | | *BRCA1* | *BRCA2* | non–*BRCA1/2* | |
| **3 affected** | **74** | **6** | **2** | **66** | **10.8%** |
| Only breast, none under 40 | 47 | 0 | 1 | 46 | 2.1% |
| Only breast, some under 40 | 15 | 1 | 0 | 14 | 6.7% |
| Breast and ovarian, none under 40 | 9 | 3 | 0 | 6 | 33.3% |
| Breast and ovarian, some under 40 | 3 | 2 | 1 | 0 | 100% |
| **4 affected** | **35** | **5** | **3** | **27** | **22.9%** |
| Only breast, none under 40 | 15 | 0 | 0 | 15 | 0% |
| Only breast, some under 40 | 7 | 1 | 0 | 6 | 14.3% |
| Breast and ovarian, none under 40 | 11 | 3 | 1 | 7 | 36.4% |
| Breast and ovarian, some under 40 | 3 | 1 | 2 | 0 | 100% |
| **>5 affected** | **39** | **5** | **8** | **26** | **33.3%** |
| Only breast, none under 40 | 6 | 0 | 0 | 6 | 0% |
| Only breast, some under 40 | 10 | 0 | 2 | 8 | 20.0% |
| Breast and ovarian, none under 40 | 9 | 1 | 0 | 8 | 11.1% |
| Breast and ovarian, some under 40 | 14 | 4 | 6 | 4 | 71.4% |
| **Total** | **148** | **16** | **13** | **119** | **19.6%** |
| Only breast, none under 40 | 68 | 0 | 1 | 67 | 1.5% |
| Only breast, some under 40 | 32 | 2 | 2 | 28 | 12.5% |
| Breast and ovarian, none under 40 | 28 | 7 | 1 | 20 | 28.6% |
| Breast and ovarian, some under 40 | 20 | 7 | 9 | 4 | 80.0% |

*© 2001 Cancer Research Campaign*

1035 unselected breast cancer patients, where all 15 cases with heavy breast cancer family history were also mutation negative (Syrjäkoski et al, 2000). Other, yet unknown susceptibility genes remain to be identified and may account for a large proportion of breast cancer families (Rebbeck et al, 1996; Serova et al, 1997; Vehmanen et al, 1997b; Ford et al, 1998; Kainu et al, 2000).

In 295 breast cancer cases with one affected 1st degree relative only one mutation (*BRCA2*, 7708 C → T) was found giving the mutation frequency of 0.3%. In this family the index patient was diagnosed at the age of 37, and her mother had died of breast cancer at the age of 40. Ovarian cancer or a young breast cancer patient diagnosed under 40 years was present in 39 families, but among these only this one mutation was found (2.6%). This suggests that mutation screening in families with only 2 affected cases is not feasible in Finland. In contrast, Goelen et al (1999) reported that *BRCA1/2* mutation testing can be done with reasonable efficiency in the Belgian population when there are 2 symptomatic family members. Prevalent founder mutations account for a large fraction of breast cancer families in Belgium (Peelen et al, 1997; Goelen et al, 1999), while *BRCA1* and *BRCA2* mutations are more rare in the Finnish population (Vehmanen et al, 1997a,b; Huusko et al, 1998). Also in studies of patients with early onset breast cancer, only a small proportion of familial risk of breast cancer has been attributed to these two genes, and the majority appears to be due to other genes (Peto et al, 1999).

### CONCLUDING REMARKS

As the screening of both *BRCA1* and *BRCA2* is very laborious and expensive, and genetic testing may be emotionally very stressful for the families, the potential mutation carrier families should be recognized as efficiently as possible to avoid unnecessary analyses of non-carriers. Studies of breast cancer patients have indicated that it may be difficult to define mutation screening criteria among women with minimal or no family history (Malone et al, 1998). Furthermore, the carrier risks associated with the mutations may be highly variable, and population-based risk estimates have indicated much lower cancer risks than those obtained from multiple-case families and, therefore, lower predictive value of cancer for a positive mutation test result (Struewing et al, 1997; Fodor et al, 1998; Thorlacius et al, 1998; Hopper et al, 1999; Warner et al, 1999). Accordingly, genetic screening would be of greatest benefit in families with high cancer risk, i.e. strong family history (Fodor et al, 1998), and a high probability of harbouring a *BRCA1* or *BRCA2* mutation. For this study, we chose families with a defined family history, and developed a model by which likelihood of carrying a *BRCA1* or *BRCA2* mutation can be estimated for each family separately. It should be a useful tool in genetic counselling and focusing the mutation analyses, and increasing thus the cost-effectiveness of the genetic screening.

### ACKNOWLEDGEMENTS

We wish to thank Minna Merikivi for her help in patient contacts and sample collection, Merja Lindfors for technical help, and the Finnish Cancer Registry for diagnostic information. This study has been supported by the Academy of Finland, The Finnish Cancer Society, The Sigrid Juselius Foundation and Clinical Research Fund of Helsinki University Central Hospital.

### REFERENCES

Berry DA, Parmigiani G, Sanchez J, Schildkraut J and Winer E (1997) Probability of carrying a mutation of breast-ovarian cancer gene BRCA1 based on family history. *J Natl Cancer Inst* **89**: 227–238

Breast Cancer Information Core, http://www.nhgri.nih.gov/Intramural_research/Lab_transfer/Bic/

Breast Cancer Linkage Consortium (1997) Pathology of familial breast cancer: differences between breast cancers in carriers of BRCA1 or BRCA2 mutations and sporadic cases. *Lancet* **349**: 1505–1510

Chang-Claude J, Becher H, Caligo M, Eccles D, Evans G, Haites N, Hodgson S, Moller P, Weber BH and Stoppa-Lyonnet D for the EC demonstration project on familial breast cancer (1999) Risk estimation as a decision-making tool for genetic analysis of the breast cancer susceptibility genes. EC demonstration project on familial breast cancer. *Disease Markers* **15**: 53–65

Couch FJ, DeShano L, Blackwood MA, Calzone K, Stopher J, Campeau L, Ganguly A, Rebbeck T and Weber BL (1997) BRCA1 mutations in women attending clinics that evaluate the risk of breast cancer. *N Engl J Med* **336**: 1409–1415

Eerola H, Blomqvist C, Pukkala E, Pyrhönen S and Nevanlinna H (2000) Familial breast cancer in southern Finland: How prevalent are breast cancer families and can we trust the family history reported by the patient? *Eur J Cancer* **36**: 1143–1148

Fodor FH, Weston A, Bleiweiss IJ, McCurdy LD, Walsh MM, Tartter PI, Brower ST, and Eng CM (1998) Frequency and carrier risk associated with common BRCA1 and BRCA2 mutations in Ashkenazi Jewish breast cancer patients. *Am J Hum Genet* **63**: 45–51

Ford D, Easton DF, Stratton M, Narod S, Goldgar D, Devilee P, Bishop DT, Weber B, Lenoir G, Chang-Claude J, Sobol H, Teare MD, Struewing J, Arason A, Scherneck S, Peto J, Rebbeck TR, Tonin P, Neuhausen S, Barkardottir R, Eyfjord J, Lynch H, Ponder BA, Gayther SA, Birch JM, Lindblom A, Stoppa-Lyonnet D, Bignon Y, Borg, A, Hamann U, Haites N, Scott RJ, Maugard CM, Vasen H, Seitz S, Cannon-Albright LA, Schofield A, Zelada-Hedman M, and the Breast Cancer Linkage Consortium (1998) Genetic heterogeneity and penetrance analysis of the BRCA1 and BRCA2 genes in breast cancer families. *Am J Hum Genet* **62**: 676–689

Foulkes WD, Brunet JS, Warner E, Goodwin PJ, Meschino W, Narod SA, Goss PE and Glendon G (1999) The importance of a family history of breast cancer in predicting the presence of a BRCA mutation. *Am J Hum Genet* **65**: 1776–1779

Friedman LS, Szabo CI, Ostermeyer EA, Dowd P, Butler L, Park T, Lee MK, Goode EL, Rowell SE and King MC (1995) Novel inherited mutations and variable expressivity of BRCA1 alleles, including the founder mutation 185delAG in Ashkenazi Jewish families. *Am J Hum Genet* **57**: 1284–1297

Gayther SA, Mangion J, Russell P, Seal S, Barfoot R, Ponder BA and Stratton MR (1997) Variation of risks of breast and ovarian cancer associated with different germline mutations of the BRCA2 gene. *Nat Genet* **15**: 103–105

Goelen G, Teugels E, Bonduelle M, Neyns B and De Greve J (1999) High frequency of BRCA1/2 germline mutations in 42 Belgian families with a small number of symptomatic subjects. *J Med Genet* **36**: 304–308

Håkansson S, Johansson O, Johansson U, Sellberg G, Loman N, Gerdes AM, Holmberg E, Dahl N, Pandis N, Kristoffersson U, Olsson H and Borg A (1997) Moderate frequency of BRCA1 and BRCA2 germ-line mutations in Scandinavian familial breast cancer. *Am J Hum Genet* **60**: 1068–1078

Hodgson SV, Heap E, Cameron J, Ellis D, Mathew CG, Eeles RA, Solomon E and Lewis CM (1999) Risk factors for detecting germline BRCA1 and BRCA2 founder mutations in Ashkenazi Jewish women with breast or ovarian cancer. *J Med Genet* **36**: 369–373

Hogervorst FB, Cornelis RS, Bout M, van Vliet M, Oosterwijk JC, Olmer R, Bakker B, Klijn JG, Vasen HF and Meijers-Heijboer H (1995) Rapid detection of BRCA1 mutations by the protein truncation test. *Nat Genet* **10**: 208–212

Hopper JL and Jenkins MA (1999) Modelling the probability that Ashkenazi Jewish women carry a founder mutation in BRCA1 or BRCA2. *Am J Hum Genet* **65**: 1771–1776

Hopper JL, Southey MC, Dite GS, Jolley DJ, Giles GG, McCredie MRE, Easton DF and Venter DJ (1999) Population-based estimate of the average age-specific cumulative risk of breast cancer for a defined set of protein-truncating mutations in BRCA1 and BRCA2. *Cancer Epidemiol Biomarker Prevent* **8**: 741–747

Huusko P, Pääkkönen K, Launonen V, Pöyhönen M, Blanco G, Kauppila A, Puistola U, Kiviniemi H, Kujala M, Leisti J and Winqvist R (1998) Evidence for founder mutations in Finnish BRCA1 and BRCA2 families. *Am J Hum Genet* **62**: 1544–1548

Kainu T, Juo SH, Desper R, Schäffer AA, Gillanders E, Rozenblum E, Freas-Lutz D, Weaver D, Stephan D, Bailey-Wilson J, Kallioniemi OP, Tirkkonen M,

Syrjäkoski K, Kuukasjärvi T, Koivisto P, Karhu R, Holli K, Arason A, Johannesdottir G, Bergthorsson JT, Johannsdottir H, Egilsson V, Barkardottir RB, Johansson O, Haraldsson K, Sandberg T, Holmberg E, Grönberg H, Olsson H, Borg Å, Vehmanen P, Eerola H, Heikkilä P, Pyrhönen S and Nevanlinna H (2000) Somatic deletions in hereditary breasty cancers implicate 13q21 as a putative novel breast cancer susceptibility locus. *Proc Natl Acad Sci USA* **97**: 9603–9608

Ligtenberg MJ, Hogervorst FB, Willems HW, Arts PJ, Brink G, Hageman S, Bosgoed EA, Van der Looij E, Rookus MA, Devilee P, Vos EM, Wigbout G, Struycken PM, Menko FH, Rutgers EJ, Hoefsloot EH, Mariman EC, Brunner HG and Van't Veer LJ (1999) Characteristics of small breast and/or ovarian cancer families with germline mutations in BRCA1 and BRCA2. *Br J Cancer* **79**: 1475–1478

Malone KE, Daling JR, Thompson JD, O'Brien CA, Francisco LV and Ostrander EA (1998) BRCA1 mutations and breast cancer in the general population: analyses in women before age 35 years and in women before age 45 years with first-degree family history. *JAMA* **279**: 922–929

Parmigiani G, Berry DA and Aguilar O (1998) Determining carrier probabilities for breast cancer susceptibility genes BRCA1 and BRCA2. *Am J Hum Genet* **62**: 145–158

Peelen T, van Vliet M, Petrij-Bosch A, Mieremet R, Szabo C, van den Ouweland AM, Hogervorst F, Brohet R, Ligtenberg MJ, Teugels E, van der Luijt R, van der Hout AH, Gille JJ, Pals G, Jedema I, Olmer R, van Leeuwen I, Newman B, Plandsoen M, van der Est M, Brink G, Hageman S, Arts PJ, Bakker MM and Devilee P (1997) A high proportion of novel mutations in BRCA1 with strong founder effects among Dutch and Belgian hereditary breast and ovarian cancer families. *Am J Hum Genet* **60**: 1041–1049

Peto J, Collins N, Barfoot R, Seal S, Warren W, Rahman N, Easton DF, Evans C, Deacon J and Stratton MR (1999) Prevalence of BRCA1 and BRCA2 gene mutations in patients with early-onset breast cancer. *J Natl Cancer Inst* **91**: 943–9

Rebbeck TR, Couch FJ, Kant J, Calzone K, DeShano M, Peng Y, Chen K, Garber JE and Weber BL (1996) Genetic heterogeneity in hereditary breast cancer: role of BRCA1 and BRCA2. *Am J Hum Genet* **59**: 547–553

Sarantaus L, Huusko P, Eerola H, Launonen V, Vehmanen P, Rapakko K, Gillanders E, Syrjäkoski K, Kainu T, Vahteristo P, Krahe R, Pääkkönen K, Hartikainen J, Blomqvist C, Löppönen T, Holli K, Ryynänen M, Butzow R, Borg A, Wasteson AB, Holmberg E, Mannermaa A, Kere J, Kallioniemi O-P, Winqvist R and Nevanlinna Heli (2000) Multiple founder effects and geographical clustering of BRCA1 and BRCA2 families in Finland. *Eur J Hum Genet* **8**: 757–763

Serova OM, Mazoyer S, Puget N, Dubois V, Tonin P, Shugart YY, Goldgar D, 1 Narod SA, Lynch HT and Lenoir GM (1997) Mutations in BRCA1 and BRCA2 in breast cancer families: are there more breast cancer-susceptibility genes? *Am J Hum Genet* ***60**: 486–495*

Shattuck-Eidens D, Oliphant A, McClure M, McBride C, Gupte J, Rubano T, Pruss D, Tavtigian SV, Teng DH, Adey N, Staebell M, Gumpper K, Lundstrom R, Hulick M, Kelly M, Holmen J, Lingenfelter B, Manley S, Fujimura F, Luce M, Ward B, Cannon-Albright L, Steele L, Offit K, Gilewski T, Norton L, Brown K, Schulz C, Hampel H, Schluger A, Giulotto E, Zoli W, Ravaioli A, Nevanlinna H, Pyrhonen S, Rowley P, Loader S, Osborne MP, Daly M, Tepler I, Weinstein PL, Scalia JL, Michaelson R, Scott RJ, Radice P, Pierotti MA, Garber JE, Isaacs C, Peshkin B, Lippman ME, Dosik MH, Caligo MA, Greenstein RM, Pilarski R, Weber B, Burgemeister R, Frank TS, Skolnick MH and Thomas A (1997) BRCA1 sequence analysis in women with high risk for susceptibility mutations: risk factor analysis and implications for genetic testing. *JAMA* **278**: 1242–1250

Struewing JP, Hartge P, Wacholder S, Baker SM, Berlin M, McAdams M, Timmerman MM, Brody LC and Tucker MA (1997) The risk of cancer associated with specific mutations of BRCA1 and BRCA2 among Ashkenazi Jews. *N Engl J Med* **336**: 1401–1408

Syrjäkoski K, Vahteristo P, Eerola H, Tamminen A, Kivinummi K, Sarantaus L, Holli K, Blomqvist C, Kainu T and Nevanlinna H (2000) Population-based study of BRCA1 and BRCA2 mutations in 1035 unselected Finnish breast cancer patients. *J Natl Cancer Inst* **92**: 1529–1531

Szabo CI and King MC (1997) Population genetics of BRCA1 and BRCA2. *Am J Hum Genet* **60**: 1013–1020

Thorlacius S, Struewing JP, Hartge P, Olafsdottir GH, Sigvaldason H, Tryggvadottir L, Wacholder S, Tulinius H and Eyfjord JE (1998) Population-based study of risk of breast cancer in carriers of BRCA2 mutation. *Lancet* **352**: 1337–1339

Vehmanen P, Friedman LS, Eerola H, Sarantaus L, Pyrhönen S, Ponder BAJ, Muhonen T and Nevanlinna H (1997a) A low proportion of BRCA2 mutations in Finnish breast cancer families. *Am J Hum Genet* **60**: 1050–1058

Vehmanen P, Friedman LS, Eerola H, McClure M, Ward B, Sarantaus L, Kainu T, Syrjakoski K, Pyrhonen S, Kallioniemi OP, Muhonen T, Luce M, Frank TS and Nevanlinna H (1997b) Low proportion of BRCA1 and BRCA2 mutations in Finnish breast cancer families: evidence for additional susceptibility genes. *Hum Mol Genet* **6**: 2309–2315

Warner E, Foulkes W, Goodwin P, Meschino W, Blondal J, Paterson C, Ozcelik H, Goss P, Allingham-Hawkins D, Hamel N, Di Prospero L, Contiga V, Serruya C, Klein M, Moslehi R, Honeyford J, Liede A, Glendon G, Brunet JS and Narod S (1999) Prevalence and penetrance of BRCA1 and BRCA2 gene mutations in unselected Ashkenazi Jewish women with breast cancer. *J Natl Cancer Inst* **91**: 1241–1247

 *© 2001 Cancer Research Campaign*
